# Fabrication and characterization of biosilver nanoparticles loaded calcium pectinate nano-micro dual-porous antibacterial wound dressings

**DOI:** 10.1007/s40204-016-0060-8

**Published:** 2016-12-02

**Authors:** Robin Augustine, Anitha Augustine, Nandakumar Kalarikkal, Sabu Thomas

**Affiliations:** 1 International and Inter University Centre for Nanoscience and Nanotechnology, Mahatma Gandhi University, Kottayam, Kerala 686560 India; 2 School of Nano Science and Technology, National Institute of Technology Calicut, Calicut, Kerala 673601 India; 3 Department of Chemistry, Bishop Kurialacherry College for Women, Amalagiri, Kottayam, Kerala 686561 India; 4 School of Pure and Applied Physics, Mahatma Gandhi University, Kottayam, Kerala 686560 India; 5 School of Chemical Sciences, Mahatma Gandhi University, Kottayam, Kerala 686560 India

**Keywords:** Silver nanoparticles, Biophytum, Biosynthesis, Pectinate, Wound dressings

## Abstract

Development of materials for medical applications using biologically derived materials by green approaches is emerging as an important focus in the present healthcare scenario. Herein the first time, we report the plant extract mediated ultra-rapid biosynthesis of silver nanoparticles using whole plant extracts of *Biophytum sensitivum*. Synthesized nanoparticles were immobilized in nano-micro dual-porous calcium pectinate scaffolds for wound dressing application. Pectinate wound dressings containing silver nanoparticles have shown excellent antibacterial property and exudate uptake capacity while being biocompatible to the human cells.

## Introduction

Skin is the largest organ of the body which performs many crucial roles for instance as a barrier against exogenous substances including pathogens and mechanical stresses (Augustine et al. 2014a). Skin is always in direct contact with the external environment which make them highly susceptible to damage and/or injury (Fuchs 2016). Thus, fast repair of the skin after an injury is necessary. Now-a-days, polymeric wound dressings were developed to act as analog of the skin by performing many of the functions of natural skin like exudate management capacity, preventing microbial invasion and thermal protection (Miraftab et al. 2003; Augustine et al. 2014b, 2015a, b; Xu et al. 2015). Hydrogels like alginate and pectinate can manage the excessive exudate produced in the wound site and can act as a thermal barrier (Lloyd et al. 1998). However, additional strategies should be adopted to prevent the bacterial invasion and colonization in the wound. Incorporation of antimicrobial agents in the wound dressing is a robust approach to overcome wound infections (Augustine et al. 2014c). Antibiotics have been tried as antibacterial agents in polymeric wound dressings to avoid bacterial colonization in the wound (Unnithan et al. 2012). Due to the bacterial drug resistance and less chemical stability of the antibiotics, relatively stable novel materials should be exploited as antibacterial agents in wound dressings.

Silver nanoparticles (AgNP) are well established for their inhibitory activity against wide range of pathogenic microorganisms (Rai et al. 2009; Augustine and Rajarathinam 2012). There are many methods for the synthesis of metallic nanoparticles (Sundaram et al. 2012). Green approaches like biological synthesis routs have been adopted to enhance the biocompatibility of produced nanoparticles (Mohanpuria et al. 2008). In such green methods, instead of chemical reducing agents biologically derived substances are used to convert silver ions to AgNP. Natural plant extracts have been emerged as biological reducing agents in green routes for the synthesis of AgNP (Saravanan et al. 2011; Chandran et al. 2006; MubarakAli et al. 2011; Mollick et al. 2015; Latha et al. 2016; Jadhav et al. 2016). The major advantage of using extracts of the plants (whole plant, leaves, roots etc.) for AgNP synthesis is that they are locally available, safe, and the increased biocompatibility of the resulting AgNPs (Park 2014). It has been reported that the phytochemicals are involved directly in the reduction of the silver ions and the formation of AgNPs (Jha et al. 2009). The main phytochemicals involved in the reduction of silver salts are flavones, terpenoids, ketones, amides, aldehydes and carboxylic acids (Prabhu and Poulose 2012). *Biophytum sensitivum* is a plant in the *Oxalidaceae* family widely distributed in India, Nepal and in other south-east Asian countries and is used for medicinal purposes (Sakthivel and Guruvayoorappan 2012). Phytochemical analysis has shown that this plant contains various medicinal biochemicals which include amentoflavone, cupressuflavone and isoorientin. Biophytum plant extract and its bioactive compounds have been known to possess antioxidant, anti-inflammatory, antibacterial, antitumor, radioprotective, antimetastatic, chemoprotective, antiangiogenic, wound healing, immunomodulatory, anti-diabetic, and cardioprotective activity (Lee et al. 2009; Sakthivel and Guruvayoorappan 2012; Wilsky et al. 2012; Lee et al. 2013). Presence of biomolecules from this medicinal plant may enhance the biological properties of synthesized nanomaterials. Our group has demonstrated that biologically synthesized AgNPs using extracts of black pepper shows superior antibacterial property (Augustine et al. 2014d, 2015c). High quality colloidal suspensions of AgNP should show relatively narrow size distributions, high uniformity in shape and excellent dispersibility to eliminate aggregation.

Pectin is a natural, linear, heterogeneous polysaccharide industrially extracted from citrus fruit peels and apple pomace (May 1990; Augustine et al. 2015d). Pectin mainly consists of d-galacturonic acid (GalA) units joined in chains by means of α (1–4) glycosidic linkages with alternating side chains of α (1–4) D-gaIactose and d-arabinose (Augustine et al. 2013). The unique gel forming ability of polyuronates in the presence of calcium ions makes them ideal for drug delivery and wound dressing applications (Augustine and Rajarathinam 2012). The divalent calcium ions and the negatively charged carboxylate groups of the polyuronates forms intermolecular crosslinks resulting in an “egg-box” structure of rigid gel networks which are relatively stable under physiological conditions. Low methoxy (LM) pectins gels in the presence of divalent cations, such as Ca^2+^ (Augustine et al. 2015d).

There are many advantages for pectinate based wound dressings like excellent exudate uptake capacity and biocompatibility. However, such dressings are highly prone to bacterial colonization and they could not prevent invasion of such pathogenic bacteria to the wound (Mishra et al. 2011; Tummalapalli et al. 2016). Incorporation of biosynthesized AgNP in the calcium pectinate (CaP) wound dressing would be a novel approach to overcome this challenge. Our previous study demonstrated that incorporation of AgNP within the range of 0.25 and 1 wt% in the polymer matrix could provide satisfactory antibacterial property to the wound dressings (Augustine et al. 2015c).

Our aim in this study is to develop a porous flexible calcium pectinate/silver nanocomposite (CaP-AgNP) hydrogel wound dressing with excellent exudate management capacity, biocompatibility and antimicrobial properties. The advantages of using this dressing are; absorption of wound exudates, prevention of wound infection, retention of optimum moisture environment, permeation of gasses and fast wound healing. The wound healing ability and antibacterial activity of AgNP can be further enhanced due to the presence of phytochemicals from Biophytum. Thus, CaP-AgNP nanocomposite membranes will function as ideal wound dressings with excellent antibacterial property and exudate uptake capacity while being biocompatible to the human body.

## Materials and methods

### Materials used

Pectin (extracted from apple, Mw 30,000–100,000, 70–75% esterification) and silver nitrate of analytical grade quality were purchased from Sigma-Aldrich was used as the starting material without further purification. Dulbecco’s modified Eagle’s medium (DMEM), fetal bovine serum (FBS), trypsin/EDTA (ethylenediaminetetraacetic acid) solution, Mueller–Hinton agar and Nutrient broth media were purchased from HiMedia, India. Biophytum whole plants were freshly collected from the agricultural field near Poothampara, Kozhikode, India.

### Synthesis of silver nanoparticles

Hundred gram of freshly collected *Biophytum* plants were cleaned in tap water, subsequently in deionized water and grinded using mortar and pestle. 100 ml of deionized water was poured into the slurry and filtered through Whatman No. 1 filter paper. Filtrate was centrifuged at 5000 rpm to completely remove the solid substances. Fresh supernatant was used for the synthesis of silver nanoparticles.

Various concentrations of aqueous solutions (1, 2, 3, 4 and 5 mM) of silver nitrate (AgNO_3_) were prepared in double distilled water and used for the synthesis of silver nanoparticles. 100 ml silver nitrate solution was taken in a round bottom flask and heated to boiling on a magnetic stirrer. Light-mediated reduction of silver nitrate was avoided by covering the flask with aluminum foil. 10 ml of the Biophytum plant extract was added drop wise into the silver nitrate solution. During this process, the solution was stirred vigorously. Within 1 min, the color change was evident (pale orange to pale red). Then, it was removed from the hotplate and stirred for 1 h to be cooled to room temperature. A small portion of the obtained nanoparticle suspensions were used for UV–Visible spectroscopic analysis. Remaining part was centrifuged at 12,000 rpm several times in deionized water and finally in alcohol to get pure silver nanoparticles.

### UV–Vis spectroscopic analysis of AgNP

The optical properties of colloidal solution of AgNP were evaluated using a Shimadzu double-beam spectrophotometer between 200 and 600 nm at a resolution of 1 nm.

### FTIR (Fourier-transform IR) analysis of silver nanoparticles

FTIR analysis of the dried AgNP samples was carried out using Perkin Elmer, Spectrum 400 spectrophotometer to ensure the formation of silver nanoparticles from silver nitrate. FTIR measurement is useful to determine the presence of bioactive molecules, which may be responsible for stabilization of AgNPs by acting as capping agents.

### X-ray diffraction (XRD) analysis of silver nanoparticles

XRD was recorded in the 2*θ* range of 20–90° using D8-Advance of Bruker (Germany), of CuKa radiation with the energy 8.04 keV and wavelength 1.54 A°. The current was 25 mA and applied voltage was 40 kV. Obtained data were compared with the ICDD PDF2 powder diffraction database (International Centre for Diffraction Data 2007).

### Transmission electron microscopic (TEM) analysis of AgNP

JEOL JEM 2100 high resolution TEM was used to image the AgNP to understand the morphology and size distribution of synthesized AgNPs. The samples for TEM analysis were prepared by air-drying drops of dilute solutions of colloidal suspensions of AgNP on carbon films. ImageJ software was used to measure the individual particle size. Particle size was measured for 50 particles for each sample, particle size distribution curves were drawn and the average particle sizes were calculated.

### Antimicrobial activity of AgNPs

The Kirby–Bauer disc diffusion method was used to determine the growth inhibition of bacteria by the synthesized AgNPs. The bacterial strains *Escherichia coli* (ATCC 12228) and *Staphylococcus aureus* (ATCC6538-P) were used as representatives of Gram-negative and Gram-positive bacteria, respectively. Both the bacteria were cultured separately in Nutrient Broth medium at 37 °C in an incubator and prepared to the turbidity equivalent of 0.5 McFarland standards (McFarland 1907). Then, 100 µl of the bacterial suspension was spread on the Mueller–Hinton agar plates. Sterile filter paper discs with 6 mm diameter (HiMedia, Mumbai) were impregnated with AgNP synthesized at various silver nitrate concentrations so that to get a final AgNP concentration of 10 µg/disc. A standard antibiotic disc was used as positive control (Ciprofloxacin, 30 µg/disc). Both the paper discs containing AgNP and the controls were then placed on the surface of the Mueller–Hinton agar culture plates which were swabbed with the bacteria. The culture plates were incubated for overnight in an incubator at 37 °C. The diameters of the inhibition zones were measured in millimeters (mm). The experiment was repeated for three times to get an average value and expressed as mean ± S.D.

### Fabrication of CaP-AgNP wound dressing

For the fabrication of biosynthesized AgNP containing porous CaP scaffolds, lyophilization technique was used. Based on the preliminary results of antibacterial activity of AgNPs, nanoparticles synthesized using 2 mM silver nitrate solution was used for incorporating in CaP scaffolds. Pectin solution (1 w/v%) was prepared by dissolving a known quantity of pectin in 50 ml deionized water with continuous stirring in a magnetic stirrer to form a transparent solution. Biosynthesized AgNP were properly dispersed in the above solution to make CaP-AgNP dressings. CaP-AgNP containing 0.25 wt% (CaP-AgNP-0.25), 0.5 wt% (CaP-AgNP-0.5) and 1 wt% (CaP-AgNP-1) of AgNP were prepared. A bare sample without silver nanoparticle incorporation was also maintained (CaP). These solutions were poured into petri dishes (5 mm thickness) and dried in hot air oven (60 °C) for overnight to form films. The films were crosslinked with 4% calcium chloride solutions (for 1 h) and washed with distilled water for several times to remove the residual CaCl_2_. The films were lyophilized individually for 48 h.

The fabricated CaP-AgNP nanocomposite scaffolds were characterized using techniques like SEM and XRD analyses (as described in the previous section). The exudate uptake capacity, antimicrobial properties and the biocompatibility of the fabricated scaffolds were also evaluated.

### Scanning electron microscopy (SEM) of CaP-AgNP dressings

A Zeiss Ultra Plus High Resolution FEG-SEM (Zeiss, Germany), operated at 4.0 kV, using an in-lens secondary electrons (SE) detector was used for the morphological analysis of the CaP and CaP-AgNP membranes. Prior to the analysis, the samples were coated with gold/platinum alloy.

### XRD analysis of CaP-AgNP dressings

XRD patterns of CaP, CaP-AgNP wound dressings were recorded in the 2*θ* range of 5°–90° using D8-Advance of Bruker (Germany) of CuKa radiation with the energy 8.04 keV and wavelength 1.54 A°. The applied voltage was 40 kV and current was 25 mA.

### Swelling study of CaP-AgNP dressings

Ability of the CaP-AgNP dressings to absorb water from phosphate buffered saline (PBS, pH-7.4) and swell themselves was studied to understand the diffusion of wound exudates into the dressing that is essential for proper exudate management in the wound bed. Previously weighed dressings were immersed in PBS solution in pre-weighed containers for known intervals of time. The PBS solution was completely discarded at specific intervals and wet weight was measured. Percentage of swelling was calculated using the formula:

$$ {\text{Percentage swelling}} = ({\text{final weight}} - {\text{initial weight}})/{\text{initial weight}} \times 100. $$

### Antimicrobial activity of CaP-AgNP dressings

In vitro antibacterial activity of CaP and CaP-AgNP dressings was evaluated by disc diffusion method according to the National Committee for Clinical Laboratory Standards (NCCLS 2001). The procedure adopted for this experiment was similar to the antibacterial testing used for AgNP except that instead of paper discs containing silver nanoparticle CaP, CaP-AgNP-0.25, CaP-AgNP-0.5 and CaP-AgNP-1 were used. The discs of lyophilized membranes were cut into 6 mm diameter and placed on the surface of the inoculated MHA plates. The plates were incubated at 35 °C overnight to get a confluent lawn of bacterial growth. Gentamicin antibiotic discs containing 10 µg/disc were used as positive controls. The sensitivity of the microorganisms to the membranes was determined by measuring the diameter of inhibitory zones on the agar surface around the discs. All the tests were carried out in triplicate. The diameters of the inhibitory zones were measured in millimeters.

### Determination of in vitro biocompatibility of the CaP-AgNP dressings

Cell viability on CaP and CaP-AgNP wound dressings were determined by MTT (3-(4,5-dimethylthiazol-2-yl)-2,5-diphenyl tetrazolium bromide) assay. L929 fibroblast cell line was obtained from NCCS Pune and was maintained in Dulbecco’s modified eagles media (HIMEDIA) supplemented with 10% fetal bovine serum (FBS) (Invitrogen) and grown to confluency at 37 °C in 5% CO_2_ (NBS, EPPENDORF, GERMANY) in a humidified atmosphere in a CO_2_ incubator. The cells were trypsinized (500 µl of 0.025% Trypsin in PBS/0.5 mM EDTA solution (Himedia)) for 2 min and passaged to tissue culture flasks in complete aseptic conditions. Scaffolds with 1 cm^2^ size were sterilized and immersed in cell free media for 24 h. Trypsinzed cells (50,000/cm^2^) were added on the surface of samples and were allowed to grow for 24 h followed by MTT assay. Briefly, the cultured cells or tissues were washed thoroughly with PBS and then incubated in MTT solution (0.5 mg/ml MTT in PBS) for 3 h at 37 °C with 5% CO_2_ supply. Subsequently, the solution was aspirated and the insoluble formazan product was solubilized with acidified iso-propanol. Incubated at room temperature for 30 min until the cell got lysed and a purple color was obtained. The optical density was then determined at 540 nm using a multi-well plate reader (LISASCAN, Erba). Percentage of cell viability was calculated using the following equation:

$$ \% {\text{ Viability}} = ({\text{OD of test}}/{\text{OD of control}}) \times 100. $$

### Statistical analysis

All the experiments were carried out in triplicate and the results were expressed as mean ± standard deviation. Statistical significance between different groups was determined by Student’s *t* test. A *P* value less than 0.05 was considered as statistically significant.

## Results

### Visible color change and UV absorbance during the formation of AgNP

**Fig. 1:**
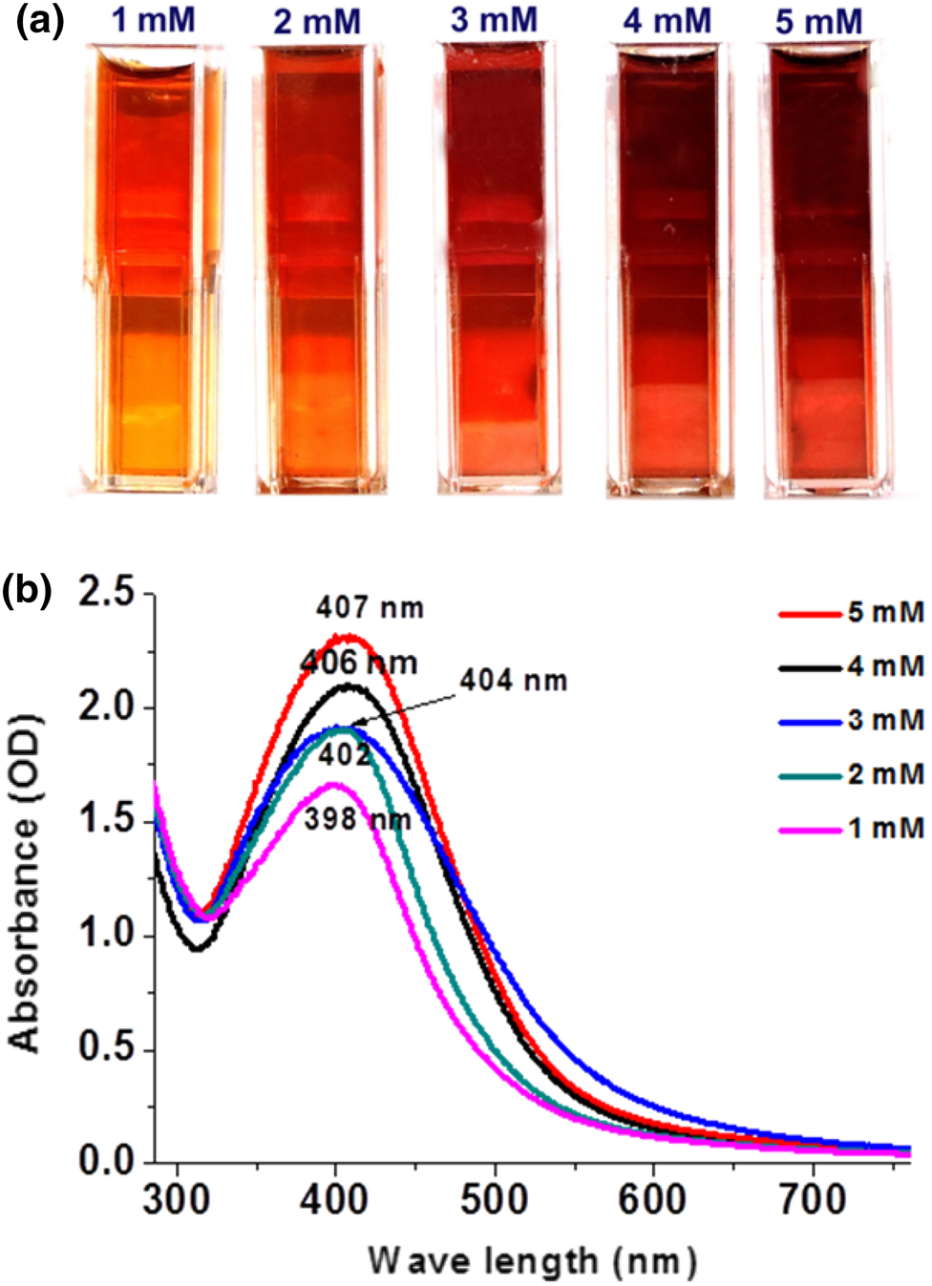
Photographic image showing the color variation of AgNPs synthesized using various concentrations of silver nitrate solutions (**a**). UV visible spectra of AgNPs synthesized using various concentrations of silver nitrate (**b**)

Visual color change will give a preliminary information regarding the formation of AgNPs. As the AgNPs are formed, the color of the solution changed from colorless to pale orange to brick red which indicates the formation of AgNPs (Fig. 1a). It is well accepted that difference in localized surface plasmon resonance (LSPR) of AgNPs with particle size makes a variation in the optical properties (Sherry et al. 2005). A very pale orange color was observed for the AgNPs synthesized at a silver nitrate concentration of 1 mM. There was a considerable increase in the redness of the solution when the concentration of silver nitrate was increased up to 5 mM. Corroborating the results of previous studies, as the concentration of silver nitrate increases, aggregation of formed silver ions occurs and which leads to the formation of larger sized AgNPs (Augustine et al. 2014c).

The UV–Visible absorption spectra of biosynthesized AgNPs are shown in Fig. 1b. Characteristic absorption maxima of silver nanoparticles can be observed in between 300 and 600 nm. For 1 mM solution, AgNPs have shown absorbance maximum at 398 nm, 2 mM solution has absorbance maximum at 402 nm, 3 mM has at 404 nm, 4 mM has at 406 nm, and 5 mM solution has at 407 nm. These range (398–406 nm) of absorption maxima indicate the presence of AgNPs with a particle size below 20 nm (Pillai and Kamat 2004). Localized surface plasmon resonance (LSPR), which is a result of collective oscillations of a nanoparticle’s conduction band electrons, is the reason for the variation in the optical properties of nanoparticles (Sherry et al. 2005). Characteristics of the surface plasmon absorption depend on the size and shape of the nanoparticles (Wang et al. 2007). The absorption maxima shift towards red with increasing molar concentration of silver nitrate which is an indication of the increase in particle size of AgNPs (Rai et al. 2006; Song and Kim 2009; Fayaz et al. 2009).

### FTIR analysis of AgNPs

**Fig. 2:**
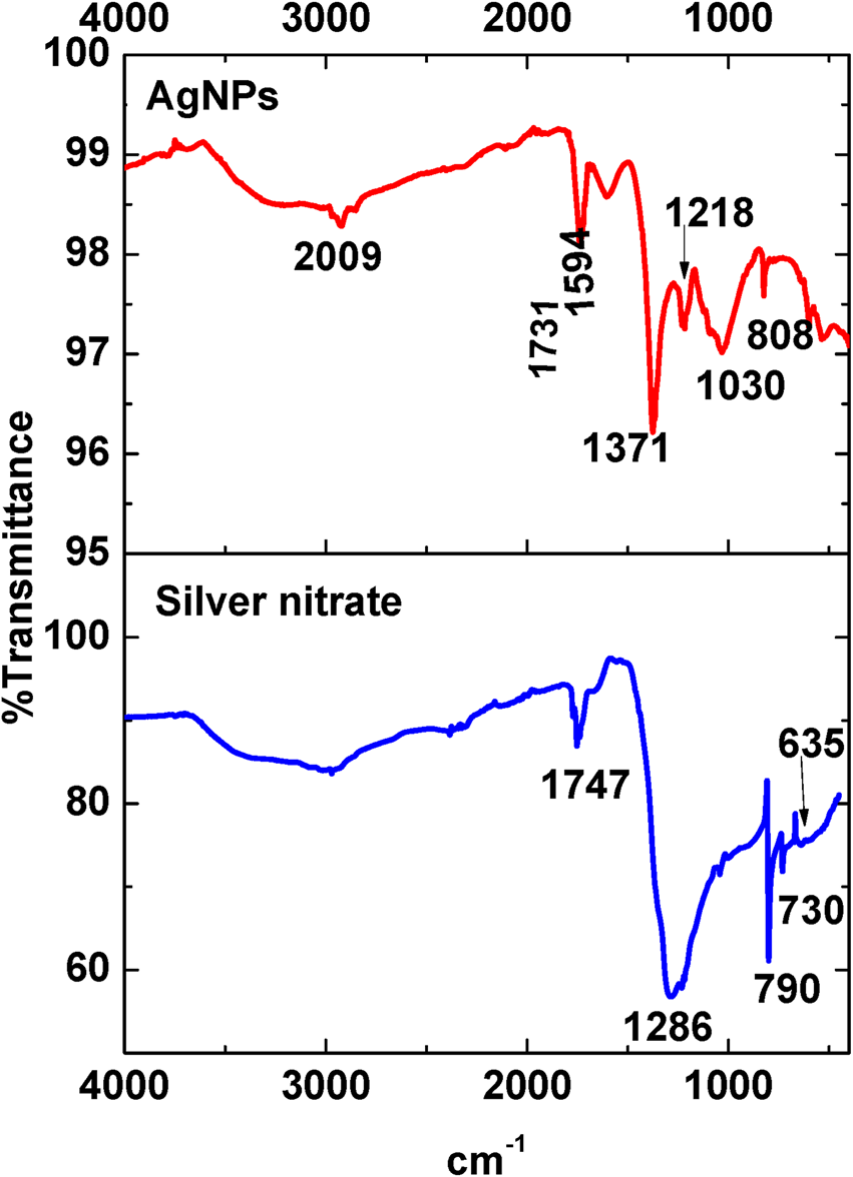
Representative FTIR spectra of biosynthesized AgNPs and silver nitrate

The FTIR spectra of unreduced silver nitrate and AgNPs after the reduction and stabilization by Biophytum extract were taken and presented in Fig. 2. FTIR analysis will help to identify the biomolecules present in Biophytum extract which was present over AgNPs. While comparing the FTIR spectra of unreduced silver nitrate and biosynthesized AgNPs, disappearance of certain peaks and appearance of some new peaks was observed. Corroborating to the previous studies, absorbance bands in pure silver nitrate were observed in the region of 450–750 cm^−1^ and were 1747, 1286, 790, 730 and 635 cm^−1^ which are due to the presence of nitro compounds (Augustine and Rajarathinam 2012, 2014c). A broad peak at 1286 cm^−1^ which was present in the spectrum of silver nitrate was not observed in the spectrum of AgNPs. It indicates the loss of nitro group from silver species during the formation of AgNPs.

The FTIR spectrum of AgNPs showed a broad peak in between 2800 and 3500 cm^−1^ and distinct peaks at 1731, 1594, 1371, 1218, 1030 and 808 cm^−1^. The broad absorption peak in between 2800 and 3500 cm^−1^ represents the presence of OH functional groups. Peak at 1731 cm^−1^ is an indication of C=O stretch and probably due to aromatic esters present in *B*. *sensitivum* (Amentoflavone, Cupressuflavone and/or Isoorientin). Ketones show their carbonyl C=O stretch at 1740–1730 cm^−1^, but also exhibit their characteristic absorption at 1300–1000 cm^−1^ from the couplings of C–O and C–C stretches. Thus, the presence of C–O stretch in between at 1218 and 1030 cm^−1^ may be due to the covalent linking of C=O groups containing flavonoids to the nanoparticles. The peak 1594 cm^−1^ is due to carbon–carbon stretching vibrations in the aromatic rings of the flavonoids attached to the nanoparticles.

These observations indicate the presence and binding of certain biomolecules with AgNPs. It may be due to the binding of one or more flavanones (Amentoflavone, Cupressuflavone, Isoorientin etc.) and/or amide-containing alkaloids which is present in Biophytum to the synthesized AgNPs. This may contribute to the stability of synthesized AgNPs (Augustine et al. 2014c).

### X-ray diffraction (XRD) analysis of AgNPs

**Fig. 3:**
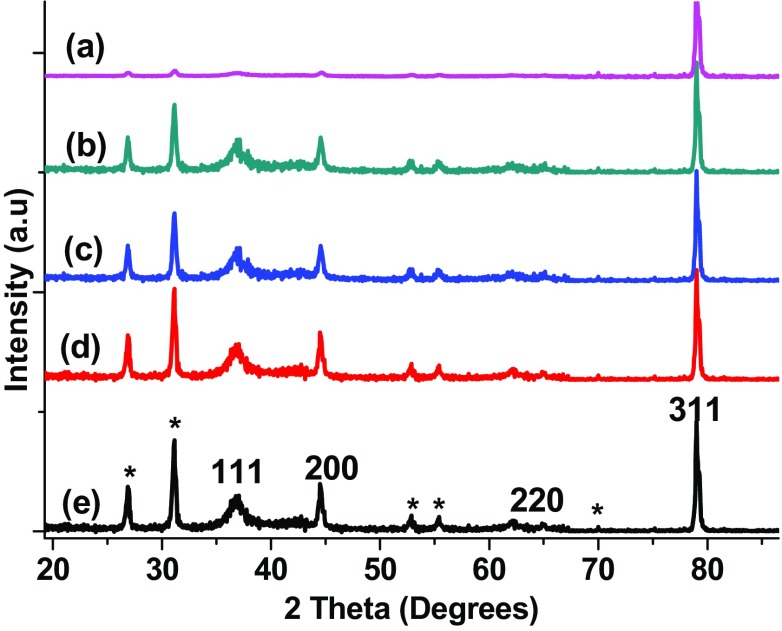
XRD patterns of AgNPs synthesized using 1 mM (*a*), 2 mM (*b*), 3 mM (*c*), 4 mM (*d*) and 5 mM (*e*) silver nitrate solutions

The structure of prepared AgNP has been investigated by X-ray diffraction (XRD) analysis. XRD patterns of the synthesized AgNPs are shown in the Fig. 3. The obtained XRD patterns have indicated the successful formation of AgNPs. The data shows diffraction peaks at 2*θ* = 38.2°, 44.05°, 64.7° and 77.8° which can be indexed to (111), (200), (220) and (311) crystalline planes of cubic silver (PDF No. 04-0783). Obtained results clearly indicate that the AgNP formed by the reduction of Ag^+^ ions by the biophytum extract are crystalline in nature. The broad nature of the XRD peaks could be attributed to the very nano size of the particles. Average particle size has been calculated using Debye–Scherrer formula:

$$ D = 0.9\lambda /\beta\,\text{Cos} \theta , $$

where ‘*λ*’ is wave length of X-ray (0.1541 nm), ‘*β* is FWHM in radians, ‘*θ*’ is the diffraction angle and ‘*D*’ is the diameter (size) of the synthesized nanoparticles. The particle size was found to be 7.62, 8.52, 8.61, 9.62 and 12.47 nm for AgNPs which are synthesized using 1, 2, 3, 4 and 5 mM silver nitrate solutions, respectively. The unassigned peaks denoted by (*) could be due to the presence of crystalline organic phases from the plant extract. Awwad et al., observed such additional peaks in the XRD spectrum of green synthesized AgNPs using carob *Olea europaea* leaf extract (Awwad et al. 2012) and carob leaf extract (Awwad et al. 2013). Sathyavathi et al. (2010) and Khalil et al. (2014) also observed such additional peaks in the XRD pattern of biosynthesized AgNPs using *Coriandrum sativum* leaf extract and olive leaf extract, respectively.

### TEM imaging of AgNPs

**Fig. 4:**
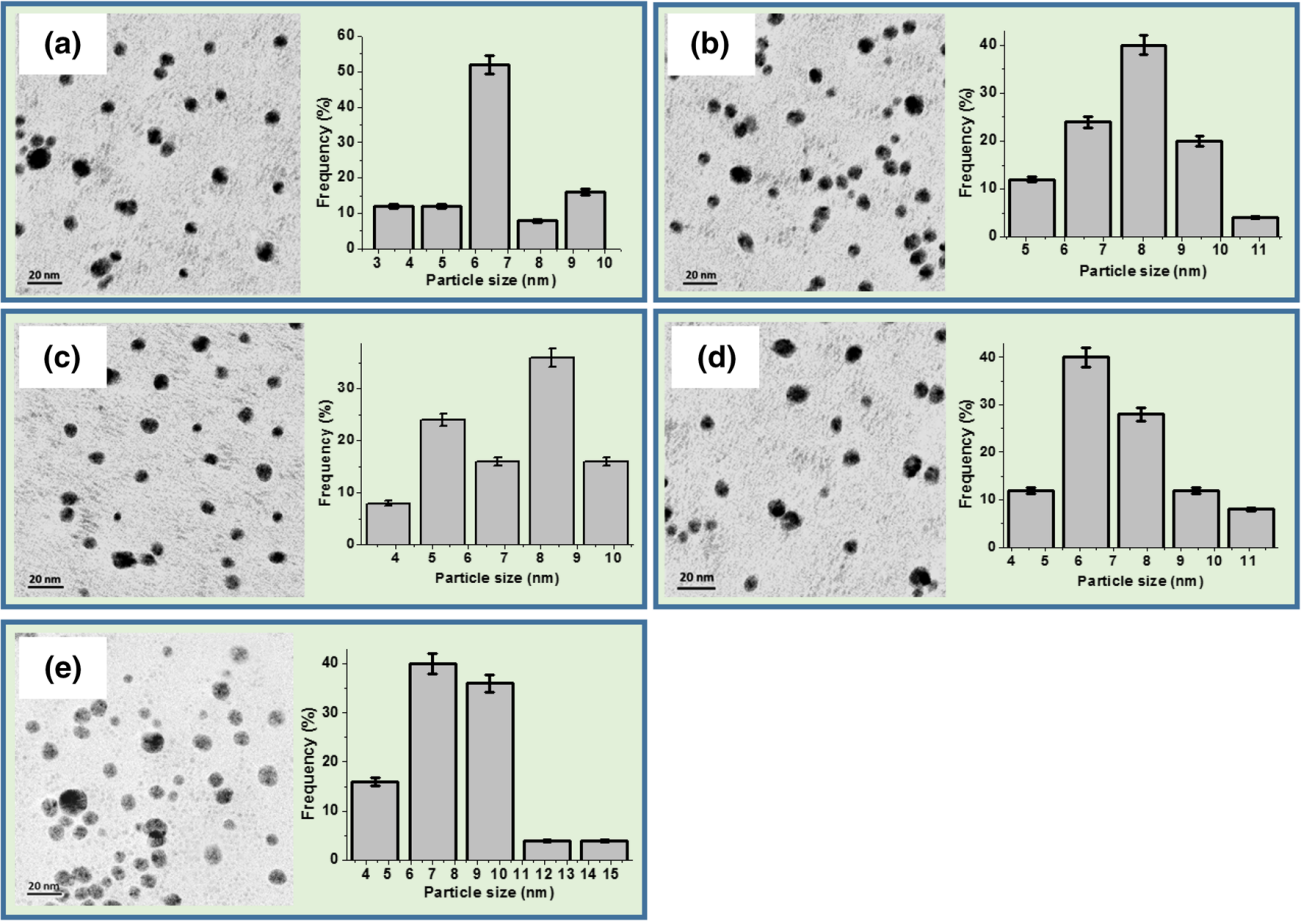
TEM images of AgNPs synthesized using silver nitrate solutions of 1 mM (**a**), 2 mM (**b**), 3 mM (**c**), 4 mM (**d**) and 5 mM (**e**). *Graphs* shows the particle size distribution of each sample based on TEM images (**b**)

TEM is a powerful tool to understand the morphology as well as particle size of nanomaterials. TEM images of AgNPs obtained at various silver nitrate concentrations are given in Fig. 4.

In general, the synthesized AgNPs were spherical in morphology without forming any agglomerates. The average particle size at 1 mM silver nitrate solution concentration was 7.4 nm. At a silver nitrate concentration of 2 mM, almost similar morphology was obtained as in the case of 1 mM solution but the average particle diameter increased to 8.2 nm (Fig. 4b). When the silver nitrate concentration was further increased, the size of the nanoparticles was also tends to increase. At 3 mM silver nitrate concentration, AgNPs with an average particle size of 8.8 nm were obtained (Fig. 4c). From Fig. 4d, it is clear that at 4 mM silver nitrate concentration, the average particle size was 9.1 nm and the morphology was still comparable with those synthesized using 1, 2 and 3 mM silver nitrate solutions. The particle size distribution was broadened for the AgNPs synthesized using 5 mM silver nitrate solution and the average particle size was 11.4 nm. Previous studies showed that small nanoparticles formed in the solution themselves can act as nucleation centers, and thus at higher concentration of metal ions these seeds will grow further and hence large sized nanoparticles will be obtained (Mallik et al. 2001). A similar mechanism could be proposed here also.

### Evaluation of antibacterial property of AgNP

The antibacterial activity of the AgNPs was evaluated by observing their inhibitory activity against both Gram-positive (*S. aureus*) and Gram-negative (*E. coli*) bacteria by disc diffusion method (Kirby–Bauer method). The results of the antimicrobial testing are shown in Table 1. From the table, it is evident that all the AgNPs has shown excellent antibacterial activity against both *S. aureus* and *E. coli*. While comparing the inhibitory zone diameters of AgNPs synthesized using 1, 2 and 3 mM silver nitrate solutions against *S. aureous* and *E. coli*, there was no considerable statistical difference (*P* > 0.1). However, the AgNPs synthesized using 1, 2 and 3 mM silver nitrate solutions showed superior antibacterial property than those synthesized using 4 and 5 mM silver nitrate solutions (*P* < 0.05) against both bacteria. Nanoparticles prepared using low concentrations of silver nitrate were more effective to inhibit both *E. coli* and *S. aureus*. This can be due to the fact that antibacterial activity of AgNPs was found to be dependent on the size of the nanoparticles and as the size increases the antibacterial activity decreases (Pana´cˇek et al. 2006). From the morphological features of the AgNPs, it was evident that AgNPs synthesized using 4 mM and 5 mM silver nitrate solutions were comparatively larger in size. All the synthesized nanoparticles have shown more antibacterial activity against *E. coli* than *S. aureus* as reported by other workers (Shrivastava et al. 2007). The reason for such an observation could be explained in terms of the difference in the cell wall structure of these bacteria.

**Table 1:** Diameter of inhibitory zone

Silver nitrate concentration (mM)	Inhibitory zone (mm)	
	*E*. *coli*	*S*. *aureus*
1	13.4 ± 1.2	11.1 ± 1.7
2	13. 9 ± 0.9	11.5 ± 1.1
3	12.5 ± 1.1	11.2 ± 0.7
4	10.8 ± 0.6	10.5 ± 1.4
5	10.5 ± 1.4	9.6 ± 0.9
Ciprofloxacin	30 ± 2.6	29.5 ± 1.8

Moreover, the biomolecules present on AgNPs enhances the antibacterial efficacy due to the antibacterial property of these molecules. Antibacterial activity of *B. sensitivum* on both Gram-negative and Gram-positive bacteria was already reported (Natarajan et al. 2010; Sakthivel and Guruvayoorappan 2012). Based on the results obtained from the disc diffusion technique, it was clear that the synthesized AgNPs can successfully inhibit bacterial proliferation and hence it can be used for the development of materials where antibacterial property is essential.

### Morphological features of CaP-AgNP membranes

**Fig. 5:**
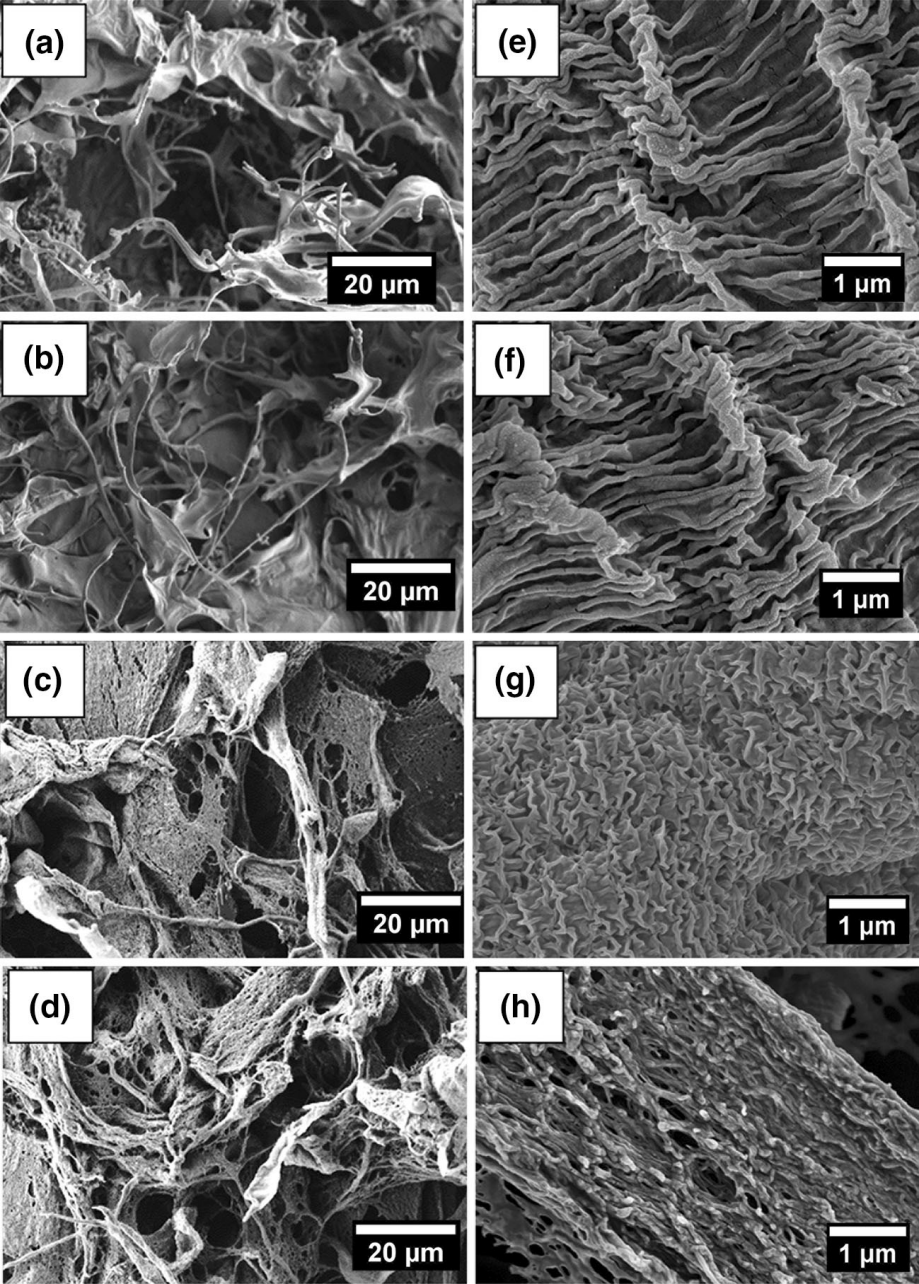
SEM images of CaP (**a**, **e**) and CaP-AgNP-0.25 (**b**, **f**), CaP-AgNP-0.5 (**c**, **g**) and CaP-AgNP-1 (**d**, **h**)

Morphological features of the fabricated dressings were evaluated by SEM analysis and given in Fig. 5. From the figure, it is clear that the fabricated scaffolds were nano-microporous in structure. This kind of dual-porous structures are advantageous for wound dressings in the sense that a large degree of porosity is required for the gas exchange and immediate swelling (Mi et al. 2001). Addition of 0.25 wt% of AgNP did not change the morphology of the dressings and were similar to the bare CaP dressings in terms of nano and microporosity. However, CaP-AgNP-0.5 and CaP-AgNP-1 showed much variation than bare CaP dressings in morphological features. They possessed more micropores and some special nanosurface architectures.

### XRD analysis of CaP-AgNP

**Fig. 6:**
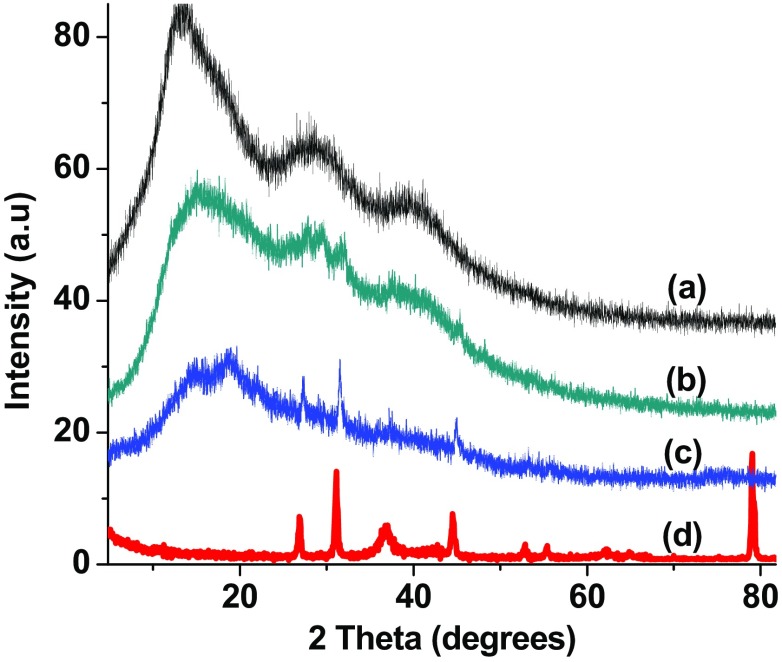
XRD spectra of bare CaP (*a*), Cap-AgNP-0.25 (*b*), CaP-AgNP-1 (*c*) and AgNP (*d*)

XRD analysis of the scaffolds confirmed the presence of AgNP in the CaP-AgNP dressings. Bare CaP dressings were relatively amorphous in nature as evident from the week peaks observed in the XRD pattern (Fig. 6). Three broad peaks were found at 2 theta 13.7, 30 and 55 degrees. These peaks are due to the egg-box junction zones of calcium pectinate (Guo et al. 2014). Li et al. (2007) also reported comparable XRD patterns of the egg-box junction zones in the case of calcium alginate. Apart from these peaks, the nanocomposites showed the characteristic XRD patterns of AgNP also. In the case of CaP-AgNP-0.25, though these patterns were present, the intensity was very low to be distinguished from the background. However, when the concentration of AgNP increased in the nanocomposites, well distinguishable sharp diffraction patterns of AgNP were observed.

### Exudate uptake capacity of CaP-AgNP

**Fig. 7:**
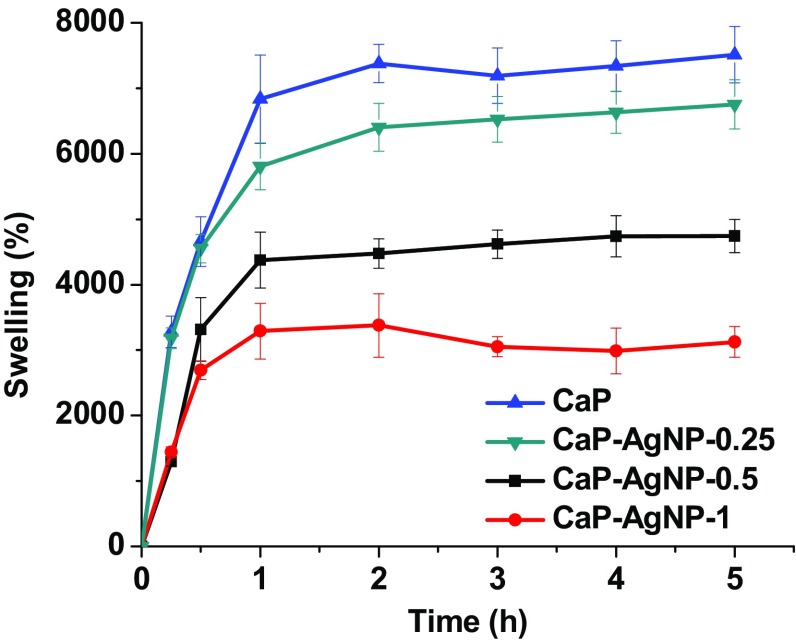
Exudate uptake capacity of CaP and CaP-AgNP-0.25, CaP-AgNP-0.5 and CaP-AgNP-1 at various time intervals in terms of percentage of swelling

The exudate uptake capacity of CaP, CaP-AgNP-0.25, CaP-AgNP-0.5 and CaP-AgNP-1 scaffolds after immersion in PBS (pH-7.4) at 37 °C up to 5 h were studied and the results are shown in Fig. 7. During the first 30 min of immersion in PBS, the swelling of the CaP and CaP-AgNP-0.25 scaffolds did not show any significant difference but both showed ~4630% of swelling (*P* > 0.05). Similarly, CaP-AgNP-0.5 and CaP-AgNP-1 scaffolds did not show any significant variation in swelling up to 30 min. However, in the case of CaP and CaP-AgNP-0.25, from 1 h of immersion period onwards, the value increased to ~6815 and ~5780%, respectively, with statistically significant difference (*P* < 0.05). At second hour of immersion, CaP and CaP-AgNP-0.25 have reached a swelling of ~7360 and ~6415%, respectively. This trend was continued even at fifth hour immersion. CaP-AgNP-0.5 and CaP-AgNP-1 showed a maximum swelling after 1 h immersion in PBS which were ~4360 and ~3280%, respectively. From 1 h onwards, all the scaffolds were shown statistically significant variation in swelling each other (*P* < 0.05). Thus, the swelling of all the scaffolds increased with increasing immersion time up to 1 h. However, a further increase in the immersion time did not produce any significant variation in swelling. There was no effect of AgNP on the swelling of CaP up to 1 h of immersion in PBS. In contrast, presence of AgNP in the CaP has a significant effect on the swelling of CaP after 1 h of immersion (*P* < 0.05). As the percentage of AgNP in the CaP was increased, the percentage of swelling was decreased. Polymers in general and especially hydrogels shows a reduction in swelling in water when nanoparticles are incorporated in them (Fan et al. 2013).

### Antibacterial property of CaP-AgNP scaffolds

**Fig. 8:**
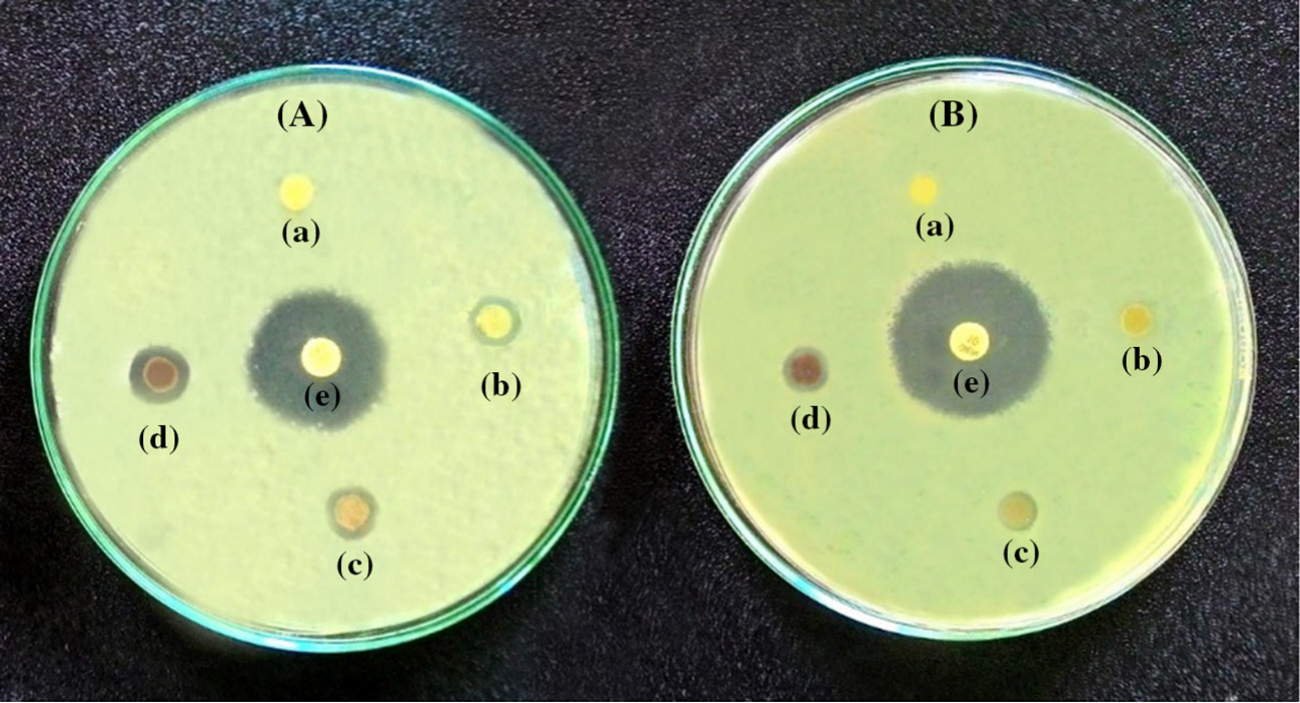
Plates showing the antibacterial activity of the fabricated CaP (*a*), CaP-AgNP-0.25 (*b*), CaP-AgNP-0.5 (*c*), CaP-AgNP-1 (*d*) against *E. coli* (*plate* **A**) and *S.* aureus (*plate* **B**). Gentamicin discs were used as the positive control (*e*)

The antibacterial activity of the CaP-AgNPs containing various concentrations of AgNP was evaluated by disc diffusion technique against a common Gram-negative (*E.coli*) and Gram-positive (*S.aureus*) bacteria and the results are shown in Fig. 8 and Table 2. From these results, it is evident that fabricated scaffolds containing AgNP have shown good inhibitory activity against both *E. coli* and *S. aureus.* The CaP membranes did not show any antibacterial activity against the tested bacteria. CaP-AgNP-0.25 has showed statistically significant antibacterial activity with an inhibitory zone diameter of 8.7 ± 0.6 against *E.coli* but it does not show any activity against *S. aureus.* Similarly, CaP-AgNP-0.5 has shown an inhibitory zone diameter of 9.3 ± 0.2 and 6.7 ± 0.5 against *E. coli* and *S. aureus*, respectively. CaP-AgNP-1 showed an inhibitory zone diameter of 11.2 ± 0.4 and 7.8 ± 0.9 against *E.coli* and *S. aureus,* respectively. The antibacterial activity of CaP-AgNP membranes was higher against *E.coli* than against *S. aureus* (*P* < 0.05). Previous studies also showed that silver nanoparticles could more effectively inhibit *E. coli* than *S. aureus* (Kim et al. 2011). This higher activity of CaP-AgNP against *E. coli* might be due to the difference in cell walls between Gram-positive and Gram-negative bacteria. Apart from inherent bactericidal property of silver nanoparticles, the presence of biomolecules over biosynthesized nanoparticles enhances antibacterial efficacy of CaP-AgNP nanocomposite membranes. Based on the results obtained from the disc diffusion technique, it is clear that the fabricated CaP-AgNP scaffolds, especially CaP-AgNP-0.5 and CaP-AgNP-1 can effectively inhibit bacterial colonization in wounds. Due to the excellent antibacterial activity, they can be used for wound dressing applications.

**Table 2:** Inhibition zone diameter from disc diffusion method using CaP and CaP-AgNP wound dressings on *E. coli* and *S. aureus*

Sample	Inhibition zone diameter (mm)	
	*E. coli*	*S. aureus*
CaP	6.00 ± 0	6.00 ± 0
CaP-AgNP-0.25	8.7 ± 0.6	6.00 ± 0
CaP-AgNP-0.5	9.3 ± 0.2	6.7 ± 0.5
CaP-AgNP-1	11.2 ± 0.4	7.8 ± 0.9
Gentamycin	24.2 ± 0.8	26.7 ± 0.5

### In vitro biocompatibility of the CaP-AgNP wound dressings

**Fig. 9:**
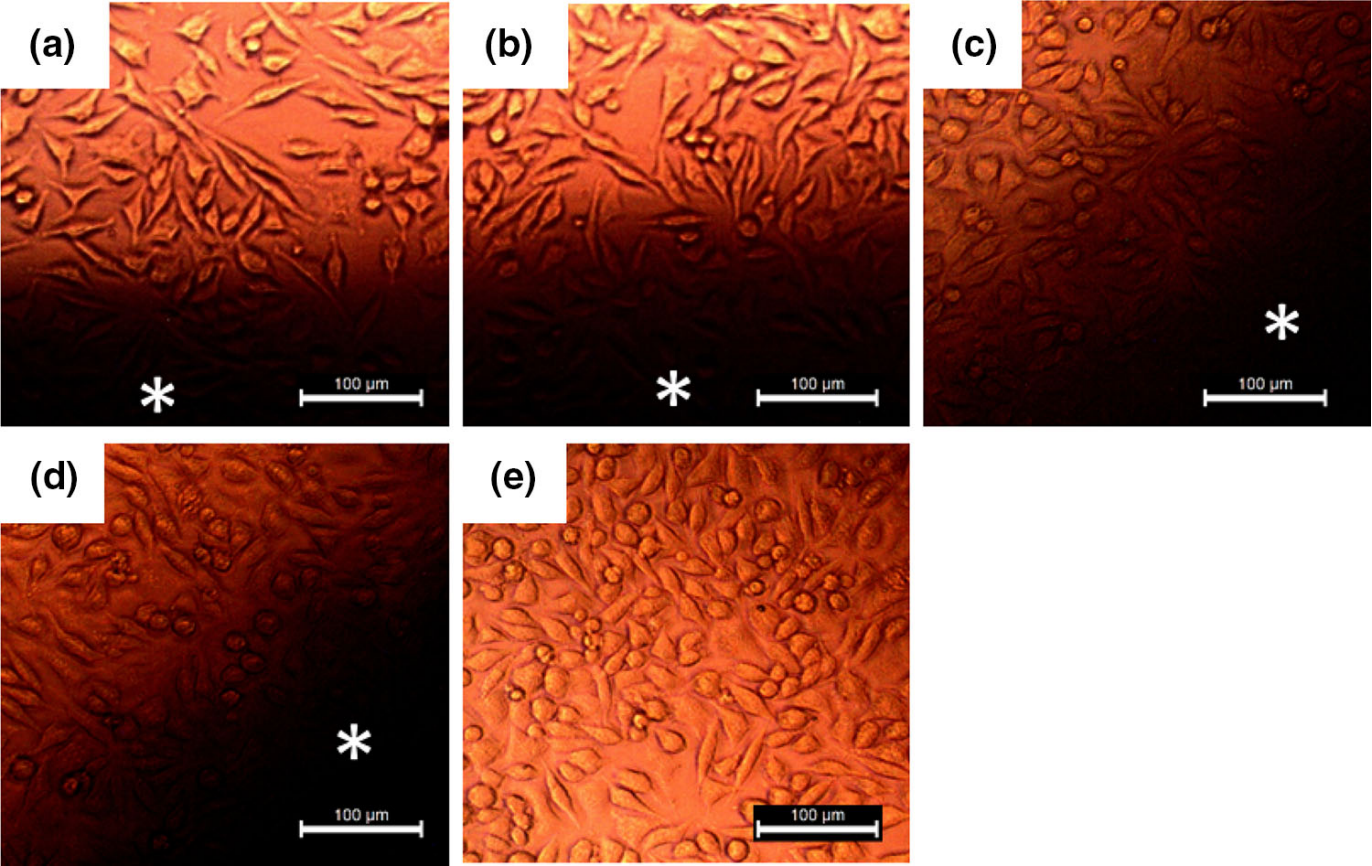
Growth of L929 fibroblast cells in the presence of fabricated scaffolds CaP (**a**), CaP-AgNP-0.25 (**b**), CaP-AgNP-0.5 (**c**), CaP-AgNP-1 (**d**) and a control plate (**e**). *Dark areas* denoted by (*asterisk*) represent the regions where the samples were placed

The bare CaP and CaP-AgNP dressings containing various concentrations (0.25, 0.5 and 1 wt%) of AgNP were evaluated for their cytotoxicity on L929 fibroblast cell lines by MTT cell viability assay. The obtained results of this study is given in Table 3. The viability of the L929 cells cultured with the bare CaP (98 ± 3) with that of the cells cultured with CaP-AgNP-0.25 (97 ± 2) and CaP-AgNP-0.5 (94 ± 4) were very close. However, CaP-AgNP-1 has shown a slight reduction in viability compared to other samples (*P* < 0.05). This corroborates the cytotoxic effects of AgNPs to impair mitochondrial function, as reported by other researchers (Burd et al. 2007; Foldbjerg et al. 2011). The relative mitochondrial activity of CaP-AgNP-1 was found to be 86 ± 7%. However, compared to the cytotoxic effects reported by other workers, materials fabricated in this study were superior in the sense that they were all below the approved toxicity level. Moreover, Fig. 9, gives direct evidence of biocompatibility of the fabricated materials on L929 fibroblast cells. Cells grown at the vicinity of the samples that contains AgNP were comparable to that of the control plates in terms of both cell morphology and cell density. Many studies indicated that the AgNPs and silver ions deleteriously affect mitochondrial functionality, and this is probably correlated to the generation of ROS (AshaRani et al. 2008). Superior biocompatibility of the CaP-AgNP scaffolds might be due to the presence of biologically derived molecules as capping agents over the nanoparticles. The viability of the cells cultured with all the CaP-AgNP scaffolds came between ~86 and ~100%, demonstrating that all the AgNP containing scaffolds were apparently nontoxic to L929 cells, indicating their biocompatibility and potential uses for wound coverage applications.

**Table 3:** Percentage of cell viability by MTT assay

Sample	Percentage of viability (%)
Control	100 ± 0
CaP	98 ± 3
CaP-AgNP-0.25	97 ± 2
CaP-AgNP-0.5	94 ± 4
CaP-AgNP-1	86 ± 7

Based on the overall performance of the scaffolds, especially the antibacterial performance and cell viability, CaP-AgNP-0.5 can be considered as the optimum candidate for future studies. This sample showed considerable antibacterial property against both Gram-negative and Gram-positive bacteria while being relatively biocompatible to human cells.

## Conclusion

In this study, we have first time demonstrated that using *Biophytum sensitivum* plant extracts, silver nanoparticles can efficiently be produced without the use of hazardous and toxic reducing agents, stabilizing agents and solvents. Silver nitrate solution was used as the precursor and aqueous extracts of the medicinal plant, *Biophytum* as reducing as well as stabilizing agent. The average particle size of the silver nanoparticles has been found to depend on the molar concentration of the silver nitrate solution used for the synthesis. The average particle size was below 10 nm unless the concentration of the silver nitrate solution was above 4 mM. FTIR analysis has shown the presence of phytochemicals that were attached on the synthesized nanoparticles. Synthesized nanoparticles were incorporated in calcium pectinate wound dressings. These wound dressings were nano and microporous in morphology and shown excellent exudate uptake capacity. They were effective against both *E. coli* and *S. aureus* while being highly biocompatible to the human cells. Based on the overall performance, calcium pectinate scaffolds containing 0.5 wt% AgNP can be considered as the optimum formulation for future studies. Incorporation of these biologically acceptable silver nanoparticles with antibacterial property in calcium pectinate scaffolds, makes this approach potentially exciting for the commercial production of greener wound dressings.
